# Transcriptome and metabolome analyses reveal the responses of brown planthoppers to RH resistant rice cultivar

**DOI:** 10.3389/fphys.2022.1018470

**Published:** 2022-09-16

**Authors:** Chunmei Li, Zhiwen Xiong, Changran Fang, Kai Liu

**Affiliations:** Guangzhou Key Laboratory for Research and Development of Crop Germplasm Resources, Key Laboratory of Green Prevention and Control on Fruits and Vegetables in South China, Ministry of Agriculture and Rural Affairs, Zhongkai University of Agriculture and Engineering, Guangzhou, China

**Keywords:** *Nilaparvata lugens*, ecological fitness, transcriptome, metabolome, interaction

## Abstract

The brown planthopper (BPH) *Nilaparvata lugens* (Stål) (Hemiptera: Delphacidae) is one of the most destructive rice pests in Asia. The application of insect-resistant rice cultivars is currently one of the principal means of controlling BPH. Understanding the physiological response mechanisms of BPH feeding on insect-resistant rice is the key for maintaining rice yield. Here, we measured the ecological fitness and analyzed the whole-body transcriptome and metabolome of BPH reared on susceptible cultivar Taichung Native 1 (TN1) and resistant cultivar Rathu Heenati (RH). Our results showed that RH significantly decreased the survival rate, female adult weight, honeydew secretion, the number of eggs laid per female and fat content of BPH. We identified 333 upregulated and 486 downregulated genes in BPH feeding on RH. These genes were mainly involved in energy metabolism, amino acid metabolism, hormone synthesis and vitamin metabolism pathways. We also detected 145 differentially accumulated metabolites in BPH reared on RH plants compared to BPH reared on TN1 plants, including multiple carbohydrates, amino acids, lipids, and some nucleosides. Combined analyses of transcriptome and metabolome showed that five pathways, including starch, sucrose, and galactose metabolism, were altered. The network for these pathways was subsequently visualized. Our results provide insights into the mechanisms of metabolite accumulation in BPH feeding on the RH rice variety. The results could help us better understand how insect-resistant rice cultivars combat BPH infestation, which is important for the comprehensive management of BPH.

## Introduction

The brown planthopper (BPH) *Nilaparvata lugens* (Stål) (Hemiptera: Delphacidae) is one of the most destructive rice pests in Asia and is highly prone to outbreaks owing to its high fecundity, remarkable adaptability to the environment, and long-distance migration. As a typical sap-sucking insect, BPH causes harm to rice by direct sucking, ovipositing, and viral transmission, such as the rice grassy stunt virus and rice ragged stunt virus (Sõgawa, 1982; Saxena and Khan, 1985; Fu et al., 2001a; Cheng et al., 2013; Xue et al., 2014; Sun et al., 2015). Chemical control is the conventional approach for controlling BPH, but it has been a controversial topic owing to the difficulty to comply with the “3R” principles and environmental pollution resulting from the excessive use of chemical insecticides (Tanaka and Matsumura, 2000; Wu et al., 2001; Gorman et al., 2008; Peng et al., 2016; Zhang et al., 2016). Owing to their effectiveness, durability, and environmental friendliness, the cultivation and application of insect-resistant rice cultivars has been earnestly regarded as an alternative method to control BPH and is currently one of the principal means of controlling BPH (Heinrichs, 1986; Velusamy et al., 1995; Cuong et al., 1997; Widawsky et al., 1998; Renganayaki et al., 2002; Jairin et al*.*, 2007; Ferrater et al., 2013; Fujita et al., 2013). Significant advancements have been made in identifying BPH resistance genes and breeding insect-resistant rice cultivars. Hundreds of insect-resistant varieties have been successfully cultivated and applied to control BPH in the field (Lin et al., 2011; Chen et al., 2012; Yue et al., 2019).

Typically, the BPH resistance level is controlled and determined by the resistance genes present in rice (Cheng et al., 2013; Fujita et al., 2013; Xiao et al., 2013). Since 1969, when International Rice Research Institute (IRRI) identified the first BPH resistance gene *Bph1* and developed the IR26 resistant variety, more than 30 BPH resistance genes, including *Bph3*, *Bph6*, *Bph9*, *Bph14*, *Bph26*, *Bph29*, and *Bph32*, have been identified and tentatively mapped on the chromosomes of rice species (Yue et al., 2019). Rice varieties with different resistance genes had varied impacts on BPH physiological metabolism, demonstrating the specificity of action mechanism (Zhu et al., 2000; Zhou and Han, 2003; Yang et al., 2006; Cheng et al., 2013; Gong and Hou, 2017; Du et al., 2018).

The effects of insect-resistant rice varieties on the ecological fitness of BPH, including survival, growth, and reproduction, have been well analyzed (Cohen et al., 1997; Qiu et al., 2010; Li et al., 2011; Gamalath et al., 2012; Liu et al., 2017; Zhang et al., 2022). Studies on the effects of insect-resistant rice varieties on the physiological metabolism of BPH have mainly focused on the alterations in BPH metabolites or genes following the ingestion of insect-resistant rice plants (Du et al., 2009; Liu et al., 2017; Yue et al., 2019; Shi et al., 2021). Yue et al. (2019) compared the metabolic responses of BPH nymphs after ingesting susceptible TN1 and resistant IR56 plant phloem. The results showed that feeding on IR56 significantly decreased the concentrations of most sugars, vitamins, and essential amino acids, while it significantly increased the concentrations of most amides, free fatty acids, and non-essential amino acids. Normal physiological activities, substance and energy metabolism, and ecological fitness are dramatically disturbed and reduced after feeding on insect-resistant rice varieties (Peng et al., 2016; Liu et al., 2017). Ji et al. (2013) sequenced the salivary gland transcriptomes of two BPH populations originating from rice varieties TN1 and Mudgo. The results revealed that genes related to metabolism, digestion, absorption, and salivary secretion may be involved in rice resistance. Individual transcriptome or metabolome analysis can only elucidate the adaptation of BPH to insect-resistant rice varieties at a specific level of metabolites or genes, whereas the biological response is typically the result of multi-process interactions including gene expression, post-translational modification, and metabolite regulation (Srivastava et al., 2013). Therefore, combining transcriptome with widely targeted metabolome analyses is required to uncover the response mechanisms of BPH to insect-resistant rice in a broad and comprehensive manner.

The resistance mechanisms of insect-resistant rice cultivars are distinct (Liu et al., 2002; Hao et al., 2008; Du et al., 2009; Tong et al., 2012; Yang and Zhang, 2016; Liu et al., 2017; Guo et al., 2018), nevertheless, the rapid development of omics technology has greatly facilitated the thorough disclosure of these mechanisms. Accordingly, recent researches have consistently provided more information on the transcriptomic and metabolic changes in the salivary glands, fat body and honeydew excretion of species (Ji et al., 2013; Yue et al., 2019), revealing the effects of insect-resistant rice cultivars on the nutritional metabolism, energy metabolism, and nutritional supply of BPH species. Most studies have focused on specific BHP tissues and excreta that are important to the defense and digestive system of the insect (Ji et al., 2013; Yu et al., 2014; Peng et al., 2016). In this study, we selected the insect-resistant rice cultivar RH and the insect-susceptible rice cultivar TN1 as the host plants and compared the whole-body transcriptomes and metabolomes of BPH reared on different host plant phloems. We analyzed differentially expressed genes (DEGs) and differentially accumulated metabolites (DAMs) to fully discover the defense mechanism employed in RH-resistant rice cultivars against BPH. A combined analysis of the metabolome and transcriptome was also conducted to elucidate the possible mechanisms that determine metabolite accumulation when BPH that fed on the RH rice variety. The results could help us better understand how resistant rice varieties combat BPH infestation, which is important for the forecasting and managing BPH impacts.

## Materials and methods

### Plant and insects materials

The experimental rice varieties, namely susceptible TN1 and insect-resistant RH cultivars were provided by the Guangdong Academy of Agricultural Sciences (Guangzhou, China) in 2019. RH rice variety contains *BPH3* and *BPH17*. *BPH3* is a cluster of three genes encoding plasma membrane-localized lectin receptor kinases (OsLecRK1-OsLecRK3), whereas *BPH17* was mapped to the short arm of chromosome 4 between RM8213 and RM5953 with unclear genes (Sun et al., 2005; Liu et al., 2015). The rice seeds were germinated for 24 h at 26°C after soaking in water for 24 h, and then sown in a soil-filled tub (2 m × 0.9 m × 0.15 m) for 7 days. Three 10-day-old rice seedlings were transplanted into experimental pots (20 cm diameter, 10 cm height), and the pots were covered with insect-proof nets. For the subsequent experiments, 45-day-old rice plants at the tillering stage were used. The experimental BPH were reared on the susceptible Huang Huazhan rice variety for more than 10 years in a greenhouse at 26 ± 1°C with 70%–90% relative humidity and a light-dark cycle of 16L: 8D. To obtain enough new (<12 h) BPH nymphs, 50 pairs of female and male BPH were placed on Huang Huazhan rice plants, and after 72 h, all BPH were removed. Once there were sufficient quantities of newly hatched nymphs, they were used in the subsequent ecological fitness measurement experiments.

### Measurement of the ecological fitness of brown planthopper reared on TN1 and Rathu Heenati

The main stem of 45-day-old TN1 and RH was placed into a test tube (2.5 cm diameter, 7.5 cm length), which was filled with 20 ml water and sealed with cotton wool, and the rice leaves were partially exposed outside the test tube. Fifteen newly hatched BPH nymphs were introduced into each test tube. Twenty biological replicates were used for each treatment group. The development of nymphs was recorded daily until adulthood, and the rice plants were replaced frequently to ensure a constant supply of fresh food. All newly emerged adults (<12 h) in each group were weighed using an electronic balance (0.01 mg) (Sartorius, Gottingen, NI, GER). A total of ninety 1-day-old short-winged BPH were collected from each rice group, placed directly into liquid nitrogen and maintained at −80°C. Three and six biological replications were used for determining the transcriptome or metabolome, respectively, with ten BPH insects in each biological replication. A pair of newly emerged BPH insects from each rice variety was placed into a test tube, which had been prepared as described above. Fifteen biological replicates were used for each treatment group. Newly hatched nymphs (<12 h) from each replicate were counted and removed until no nymphs hatched for three consecutive days. Unhatched eggs were counted by dissecting rice plants. The total number of eggs laid was the sum of the number of nymphs and unhatched eggs. All insects were reared in a greenhouse at 26 ± 2°C, 70%–90% relative humidity, and a light-dark cycle of 16L: 8D.

### Measurement on brown planthopper honeydew excretion and total fat

BPH honeydew excretion was measured according to the methods described by Yue et al. (2019) and Liu et al. (2020). Experimental sachets (5 cm length, 3 cm width) were made using parafilm membranes and weighed using a balance (0.01 mg) (Sartorius, Gottingen, NI, GER). Two newly emerged BPH adults were placed in each sachet, and the sachets were fixed to the stem of the 45-day-old rice of different varieties 2 cm above ground level. The survival of the isolated BPH pairs was recorded daily, and the weight of the sachets in which the BPH pairs remained alive after 72 h was recorded. The changes in the weights of the sachets were used to calculate the honeydew excretion of BPH that were reared on different rice varieties. Sixty replications were performed for each treatment group.

Total fat content in BPH was measured according to the methods described by Pang et al. (2017) and Yue et al. (2019). An electronic balance was used to measure the weight of five BPH adults in each sample. Six independent samples from each treatment group were analyzed.

### RNA-sequencing and differential expression analysis

All BPH samples used in this experiment were 1-day-old short-winged BPH adults. These adults had been reared on TN1 or RH rice plants. Total RNA was extracted using TRIzol Reagent (Magen, Guangzhou, China), according to the instructions provided by the manufacturer. The integrity and quality of total RNA were assessed using an Agilent 2100 Bioanalyzer and an RNA 6000 Nano LabChip Kit (Agilent Technologies, Santa Clara, CA, United States). Transcriptome sequencing libraries were prepared according to a previously described method (Su et al., 2012). RNA (1 μg) was cleaved into short fragments and then reverse-transcribed to cDNA using the HiScript®III RT SuperMix for qPCR (+gDNA wiper) (Vazyme Biotech Co., Ltd., Nanjing, China) according to the instructions provided by the manufacturer.

Three repeats of each treatment were performed for Illumina RNA sequencing. The Illumina HiSeq™ 4,000 sequencing platform (Illumina, San Diego, CA, United States) was used to sequence the cDNA library. Adapter sequences, low-quality reads (i.e., reads with a ratio of ambiguous N nucleotides greater than 5%), and empty reads were filtered out using Cutadapt software v1.16 with parameters of—discardtrimmed-n3-e0.1 (Martin, 2011). Hisat2 software was used to map the acquired clean reads to the *N. lugens* genome (accession no. GCF_000757685.1) (Kim et al., 2015). StringTie was used to calculate fragments per kilobase of exon per million fragments mapped (FPKMs) of coding genes in each sample. Differential expression analysis of two groups was performed using the DESeq2 R package (Love et al., 2014). The differentially expressed genes (DEGs) between BPH reared on TN1 or RH rice plants were estimated using DESeq2 with a threshold of |log2 ratio| > 1 and an adjusted *p* < 0.05. GO annotation of the DEGs was performed using the Blast2GO software in the Gene Ontology database (http://geneontology.org/). The Kyoto Encyclopedia of Genes and Genomes (KEGG) Automatic Annotation Server was used to annotate DEGs in the KEGG database (http://www.genome.jp/kegg/). GO function and KEGG pathway enrichment of the DEGs were analyzed using TBtools software (Chen C. J. et al., 2016).

### Validation of differentially expressed genes by qPCR

Fifteen genes were selected for qPCR validation based on their differential expression (Supplementary Table S1). The total RNA (1 μg) was used to synthesize first-strand cDNA using the PrimeScript^™^ RT reagent kit (Takara Bio, Inc., Otsu, Shiga, Japan). qPCR assays were performed using a Light Cycler 480 System (Roche Diagnostics, Basel, Switzerland) and the ChamQ Universal SYBR qPCR Kit (Vazyme Biotech Co., Ltd., Nanjing, China) according to the instructions provided by the manufacturer. A 10 μl reaction mixture containing 1 μl cDNA, 0.3 μl each of 10 μmol L^−1^ forward and reverse primers, 3.4 μl ddH_2_O, and 5 μl ChamQ Universal SYBR qPCR master mix (Vazyme Biotech Co., Ltd., Nanjing, China) was set up. The PCR amplification conditions were as follows: 5 min at 95°C, followed by 45 cycles of 95°C for 10 s, 60°C for 20 s, and 72°C for 20 s. For each sample, three biological and three technical replicates were performed. Gene expression levels were normalized to the expression levels of *N. lugens β*-actin (Chen et al., 2013). Changes in gene expression were calculated using the 2^−∆∆Ct^ method (Livak and Schmittgen, 2001), and the results were expressed as the mean ± SE. The variations in gene expression levels across treatments were analyzed using *t*-tests in the SPSS 18.0 statistical software (*p* < 0.05). Supplementary Table S1 lists the primers used in the qPCR experiments.

### Metabolite extraction and GC–MS spectrometry

For metabolite extraction, 10 female BPH adults collected from each treatment group were weighed, placed in a 1.5 ml Eppendorf tube, and the BPH surface was rinsed with sterile water for 1 min. Then, the BPH samples were ground into a powder using an electronic grinder (TIANGEN Biological Technology Co. Ltd., Beijing, China). The recovered extract solution (1,000 μl, acetonitrile: methanol: water D 2:2:1) was added to the ground sample, which was then vortexed for 30 s. The samples were sonicated for 15 min in an ice bath before being centrifuged at 12,000 rpm for 15 min at 4°C. For analysis, the supernatant was transferred to a new Eppendorf tube. The quality control sample was prepared by mixing an equal aliquot of supernatant from each sample with 60 μl of a ribitol aqueous solution. The extract was concentrated to dryness under nitrogen stream and then redissolved in pyridine using 70 μl of methoxy-aminohydrochloride (Sigma-Aldrich). After 15 s of vortexing, the mixture was incubated on a shaker at 37°C with a rotational speed of 200 r/min for 1.5 h. The mixture was treated with 100 μl N-methyl-N-(trimethylsilyl) trifluoroacetamide (MSTFA, Sigma-Aldrich) and stored at 37°C for 30 min, followed by centrifugation at 13,000 × g for 3 min. The supernatant was collected in autosampler vials (2 ml, Agilent Technologies) for subsequent analysis. Three blank samples with methoxy-amino-hydrochloride in pyridine (70 μl, 20 mg/ml) and MSTFA (100 μl) were handled in the same manner as the controls. There were six biological replications for each BPH treatment.

The BPH compounds in the blank and test samples were separated and characterized using a DB-5ms UI column (30 m length, 25 mm internal diameter) on an Agilent 7890A GC outfitted with an Agilent 5975C VL MSD detector (Agilent Technologies, Santa Clara, CA, United States). The remaining steps of this procedure were identical to the previously described method (Yue et al., 2019).

### Spectra processing for *GC–MS*

We used AMDIS to deconvolute and calibrate the acquired mass spectra for the obtained metabolic data. To avoid false positives, peaks with areas less than 100,000 were excluded (Zhang et al., 2018). The retention time was corrected, and the BPH compound peaks were normalized using an internal ribitol standard. In addition, the factitious peaks were removed by comparing the test samples with blank samples. BPH metabolites were identified by searching the National Institute of Standards and Technology library (NIST, 8.0) using the following criteria: match value ≥750, reverse match value ≥800, and probability ≥60% (Zhu et al., 2016). Finally, the relative percentage of each BPH compound in each sample was normalized based on the quality and peak area of the added ribitol internal standard, which was subjected to multivariate statistical analyses. By mapping the differential metabolites of BPH to their biochemical pathways using the MetPA online tools (http://metpa.metabolomics.ca/), the differentially expressed metabolic pathways in different treatments could be obtained.

### Integrated analysis

To investigate the correlation between DEGs and DAMs, we conducted a correlation analysis of the transcriptome and metabolome. First, overlapping pathways between the transcriptome and metabolome were identified, and the corresponding KEGG xml files (KGML) were downloaded from the KEGG pathway database. Then, a custom Perl script was employed to estimate the interactions between these pathways, with KGML files serving as inputs (Chen D. et al., 2016; Pang et al., 2018). Finally, a network map of the transcriptome and metabolome pathways was generated using Cytoscape.

## Results

### The Rathu Heenati resistant rice variety weakened the ecological fitness of brown planthopper

**FIGURE 1:**
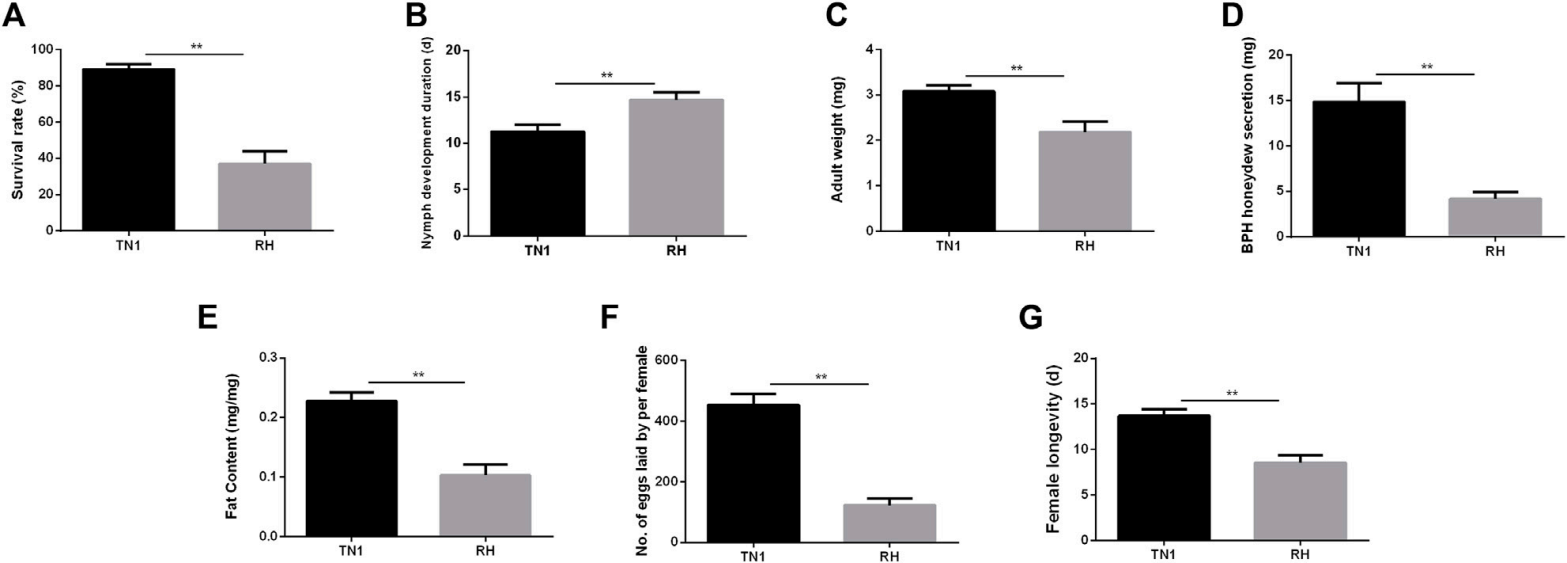
The ecological fitness of the brown planthopper (BPH), *Nilaparvata lugens* that fed on different rice varieties. **(A)** Survival rate. **(B)** Nymph development duration. **(C)** Female adult weight. **(D)** BPH honeydew secretion. **(E)** Fat content. **(F)** Number of eggs laid per female. **(G)** Female longevity. **(A–G)** were repeated for 20, 60, 6, and 15 times, respectively. The values are presented as the mean ± SEM. Asterisks indicate significant differences revealed by Student’s t test, at *p < 0.05 and **p < 0.01, respectively.

Compared to that of TN1, feeding on the RH-resistant rice varieties significantly decreased the survival rate of nymphs from 89.08% to 36.92% (*t* = 15.539, *df* = 8, *p* < 0.001; Figure 1A) and prolonged the duration of nymph development from 11.26% to 30.4% (*t* = -6.786, *df* = 8, *p* < 0.001; Figure 1B). The lower adult weight (TN1: 3.08 mg/female, RH: 2.18 mg/female, *t* = 7.661, *df* = 8, *p* < 0.001; Figure 1C) of BPH reared on the RH variety indicated that RH was not suitable for BPH nymph growth, as did the decreased honeydew excretion from 14.82 to 4.18 mg per female (*t* = 10.821, *df* = 8, *p* < 0.001; Figure 1D) and total fat content from 0.228 mg/mg to 0.103 mg/mg (*t* = 12.028, *df* = 8, *p* < 0.001; Figure 1E). There were substantial variations in fecundity between the two groups: BPH fed on TN1 laid 453.26 eggs per female, which was significantly greater than that of BPH fed on RH, which produced 122.28 eggs per female (*t* = 17.170, *df* = 8, *p* < 0.001; Figure 1F). In addition, feeding on the RH significantly shorten the female longevity from 13.7 d to 8.54 d (*t* = 10.455, *df* = 8, *p* < 0.001; Figure 1G).

### Differences in the transcriptomic profile between brown planthopper reared on TN1 and Rathu Heenati

**FIGURE 2:**
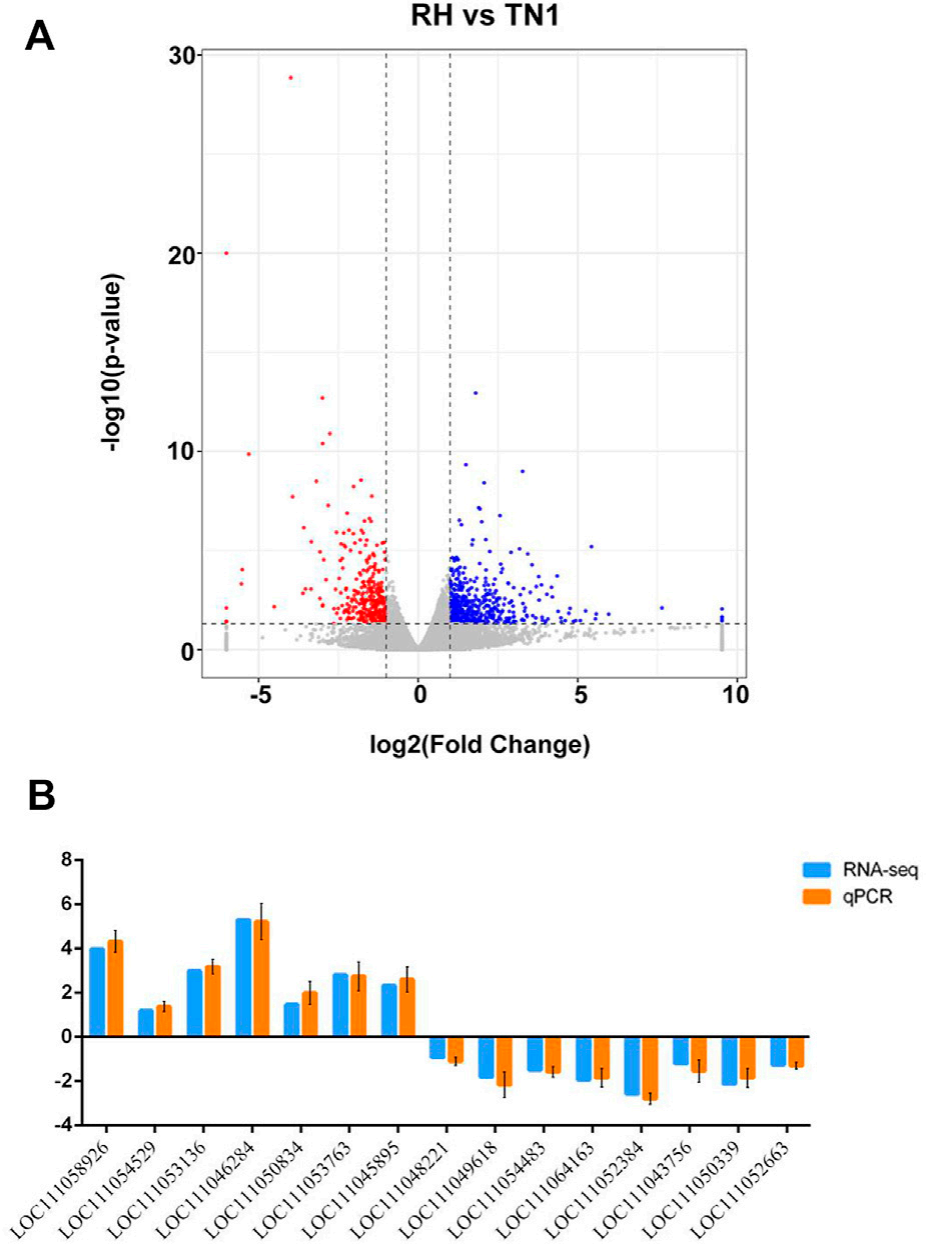
Identification of differentially expressed genes (DEGs) between BPH reared on RH and TN1 varieties. **(A)** Volcano plot for the expression pattern of each gene. Black, red, and blue points represent no difference in expression, upregulated genes, and downregulated genes, respectively. **(B)** Comparison of the transcriptome analysis and qRT-qPCR validation. LOC111058926, procollagen C-endopeptidase enhancer; LOC111054529, mitochondrial; LOC111053136, endocuticle structural glycoprotein; LOC111046284, insulin-like peptide; LOC111050834, uncharacterized protein; LOC111053763, acetylcholine receptor; LOC111045895, keratin-associated protein; LOC111048221, ATP-citrate synthase; LOC111049618, maltase; LOC111054483, sodium channel protein; LOC111064163, heat shock protein; LOC111052384, pistil-specific extensin-like protein; LOC111043756, uncharacterized protein; LOC111050339, uncharacterized protein; LOC111052663, putative serine protease. The values are presented as the mean ± SEM (*n* = 3).

The RNA-seq experiments yielded 44,782,998 and 46,395,603 raw paired-end reads for BPH adults reared on TN1 and RH varieties, with average Q30 values of 92.54% and 92.68%, respectively (Supplementary Table S2). After cleaning and quality filtering, such as removing low-quality and adaptor sequences, each sample yielded 42,248,914 and 43,400,493 high-quality clean reads. The proportion of total mapped reads to total clean reads in the *N. lugens* genome was 82.2% and 81.0% for each of the samples, respectively, and the mapped reads were used for subsequent analysis. There were 819 DEGs (4.47% of the total detected genes in the *N. luges* genome), including 333 genes upregulated and 486 genes downregulated in BPH reared on the RH variety than in BPH reared on the TN1 variety (Figure 2A; Supplementary Table S3). qRT-PCR analysis of 15 randomly selected DEGs confirmed the validity of the RNA-seq results (Figure 2B).

**FIGURE 3:**
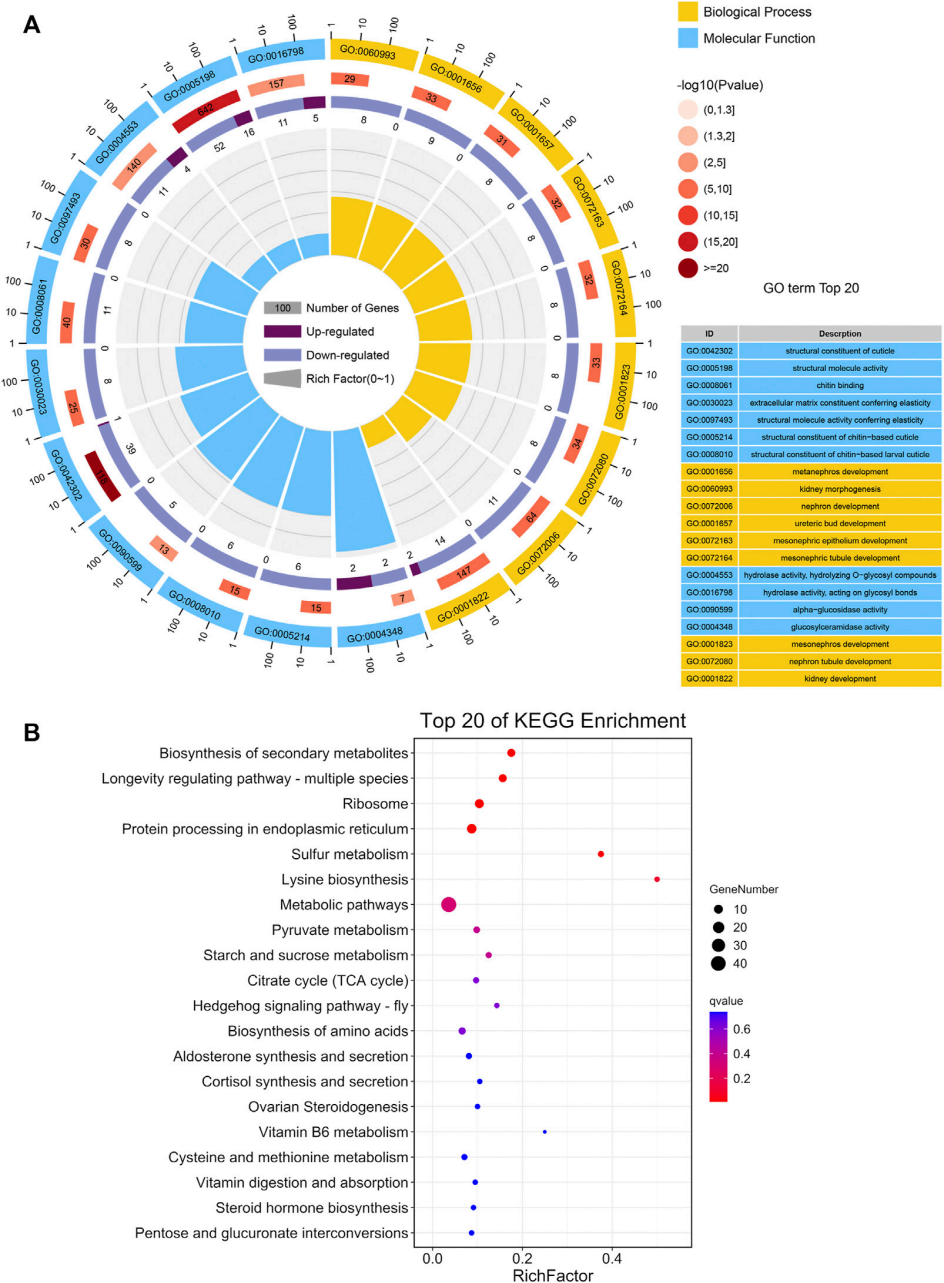
Functional CLassification of DEGs between BPH reared on RH and TN1 varieties. **(A)** GO enrichment analysis of the DEGs. The outer ring represents the number of the top 20 enriched GO terms, and different colors indicate different ontologies. Ring 2 represents the gene counts in the whole genome background, and the color changes according to the Q-value of the enrichment analysis. Ring 3 represents the counts for upregulated and downregulated genes in corresponding GO terms. Ring 4 indicates the rich factor (the number of DEGs divided by the number of background genes in the corresponding term). **(B)** The DEGs in the Kyoto Encyclopedia of Genes and Genomes metabolic pathways.

GO analysis revealed that the DEGs were significantly enriched in biological process and molecular function groups, with the most prominent functional categories being the structural constituent of cuticle, structural molecule activity, hydrolase activity, hydrolyzing O-glycosyl compounds, and glucosylceramidase activity (Figure 3A; Supplementary Table S3). The most enriched functional groups of downregulated genes included nephron development, ureteric bud development, chitin binding, and alpha-glucosidase activity, whereas the most enriched functional groups of upregulated genes included structural molecule activity, hydrolase activity, acting on glycosyl bonds and hydrolase activity, and hydrolyzing O-glycosyl compounds (Supplementary Table S4).

Additionally, KEGG analysis of DEGs was performed. The enrichment of DEGs occurred in several carbohydrate metabolism pathways, including the citrate cycle (TCA cycle), pyruvate metabolism, pentose and glucuronate interconversions, and starch and sucrose metabolism (Figure 3B; Supplementary Table S5), indicated that feeding on insect-resistant rice varieties affected the carbon utilization and energy metabolism of BPH. This is consistent with the GO enrichment of alpha-glucosidase activity and glucosyl ceramidase activity in the functional groups. Additionally, we observed that the DEGs greatly altered the longevity regulating pathway—multiple species. This was consistent with the conclusion that the longevity of adult BPH that fed on TN1 was longer than the longevity of adult BPH that fed on RH in biological assays. Moreover, lysine biosynthesis, biosynthesis of amino acids, and cysteine and methionine metabolism were observed to be enriched with DEGs, suggesting that consuming the RH rice variety impacted protein synthesis in BPH.

### Metabolic differences between brown planthopper that fed on TN1 and Rathu Heenati plants

**FIGURE 4:**
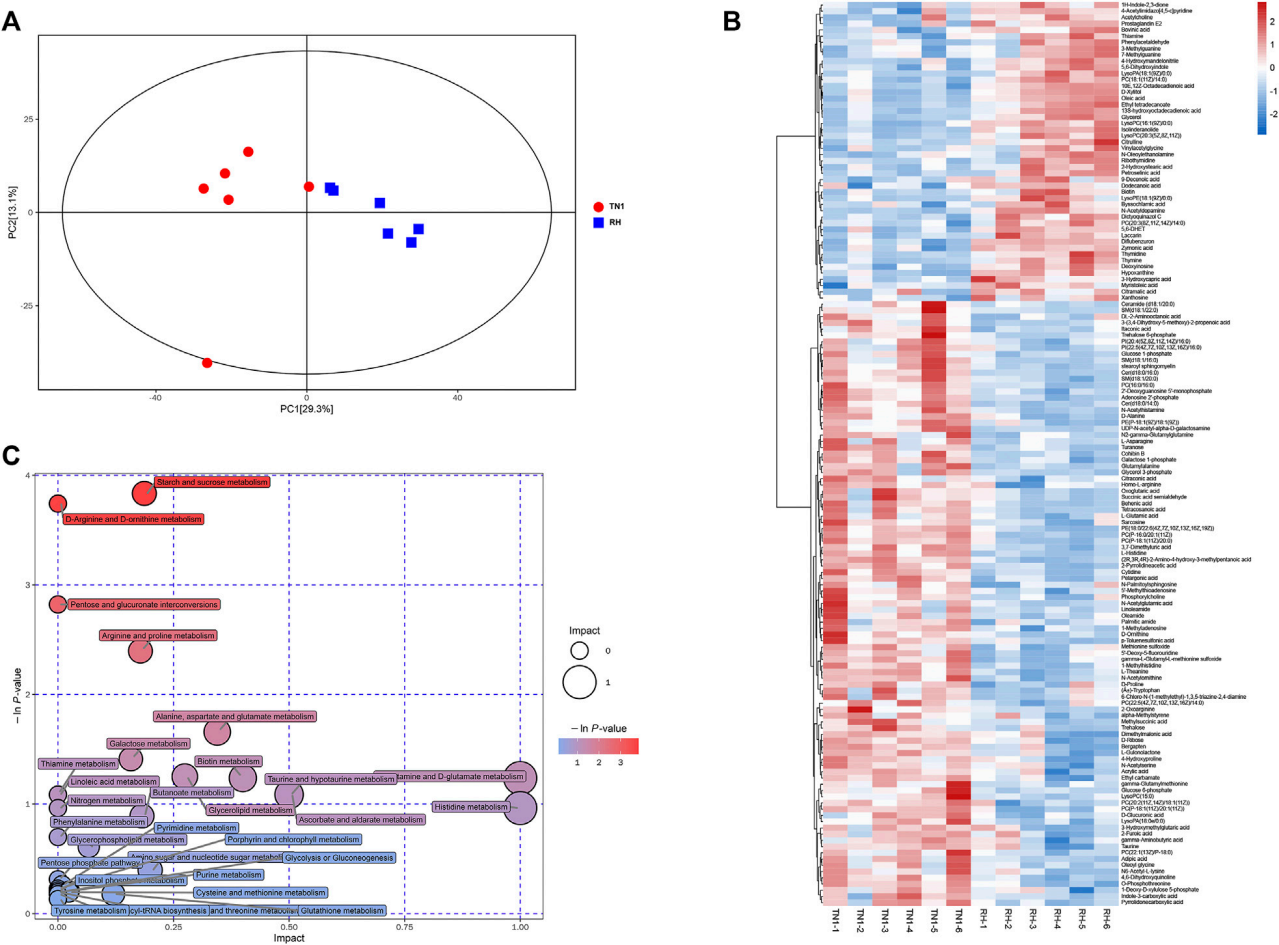
Impact of feeding different rice varieties on the metabolic profile of BPH. **(A)** Principal component analysis (PCA) plots of the metabolite composition of samples from BPH reared on RH and TN1 varieties. Each symbol represents a sample, different symbol shapes denote different groups. The circular line indicates the 95% confidence interval (Hotelling’s T-squared ellipse). **(B)** Hierarchical cluster of 12 BPH samples from the two different rice plants (*n* = 6) and heatmap displaying the levels of the differential metabolites in each sample. **(C)** KEGG pathway annotation of differential metabolites between BPH reared on RH and TN1 varieties. The point size represents the significant compound number in the corresponding pathway. 12 BPH samples from the two different rice plants (*n* = 6). The heatmap displays the levels of the differential metabolites in each sample.

n the current study, LC-MS/MS methods were used to measure the metabolome of BPH reared on TN1 and RH rice varieties. A total of 481 metabolites were detected across all samples (Supplementary Table S6). PCA analysis based on the PLS-DA model showed that these samples were clustered into two distinct groups, and the first two principal components (PC1, 29.3%; PC2, 13.1%) accounted for 42.4% of the total variation (Figure 4A), which had a high value in explaining the variation and predictive ability. The results indicated that consuming RH-resistant rice varieties had significant effects on the physiological metabolism of BPH, and the OPLS-DA score plots verified this difference (Supplementary Table S2).

We detected 145 differentially accumulated metabolites (DAMs) (Mann-Whitney *U*-test, *p* < 0.05, Figure 4B), of which 48 were upregulated and 97 were downregulated in BPH that fed on RH plants than in BPH that fed on TN1 plants. Several metabolites, including multiple carbohydrates, amino acids, lipids, and their derivatives, exhibited significant changes between the two sample groups. The contents of sugars (trehalose), glycolysis intermediates (glucose 6-phosphate), some essential amino acids (such as tryptophan, methionine, and threonine), organic amines (such as palmitic amide, asparagine, and glutamyl alanine), fatty acids (linoleamide), and certain non-essential amino acids (such as proline, histidine, and ornithine) in BPH that fed on RH plants were significantly decreased, whereas some nucleosides, such as thymine, deoxyinosine, ribothymidine, and 3-methylguanine were significantly increased (Figure 4B).

We investigated the functions of DAMs in BPH following feeding on TN1 and RH plants using KEGG pathway annotation. Comparing BPH that fed on RH plants and BPH that fed on TN1 plants, we discovered that the up-DAMs were considerably enriched in linoleic acid metabolism, phenylalanine metabolism, thiamine metabolism, and purine metabolism, whereas the down-DAMs were significantly enriched in starch and sucrose metabolism, D-arginine and D-ornithine metabolism, galactose metabolism, glycolysis or gluconeogenesis, and histidine metabolism (Figure 4C; Supplementary Table S7). These findings indicated that some primary metabolism and energy metabolism were significantly affected in BPH after having fed on RH plants, which was consistent with the transcriptomic results.

### Combined transcriptomic and metabolomic analysis

**FIGURE 5:**
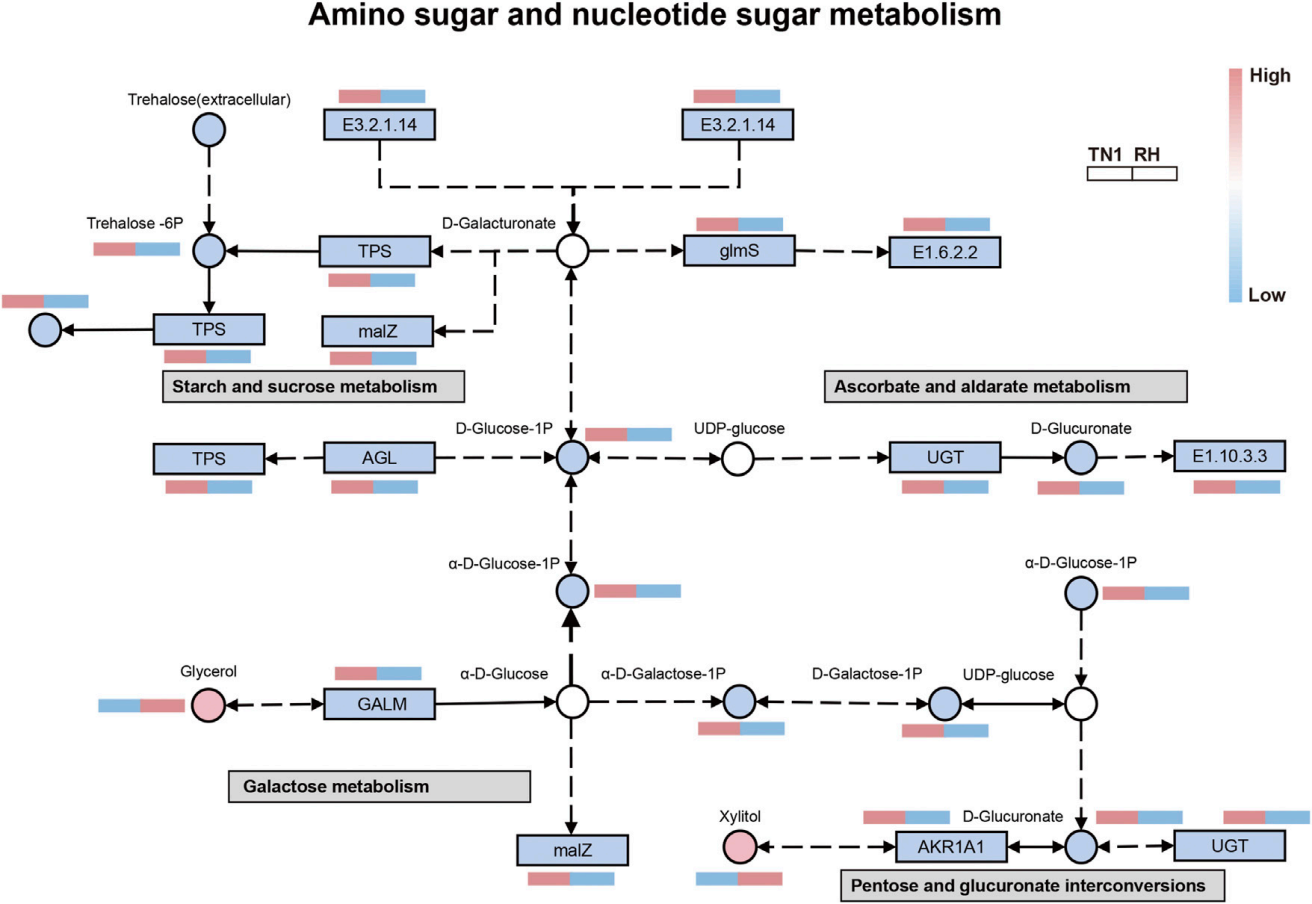
Integrated analysis of regulated pathways identified by transcriptomic and metabolomic analyses. Rectangle and circles represent genes and metabolites, respectively. For genes and metabolites, blank, red and blue indicate no difference in expression, upregulated factors and downregulated factors, respectively.

To further comprehend the correlation between the transcriptome and metabolome, an integrated analysis of the differentially expressed genes and metabolites was conducted. Five pathways were identified to be altered, with amino sugar and nucleotide sugar metabolism, starch and sucrose metabolism, ascorbate and aldarate metabolism, galactose metabolism, and pentose and glucuronate interconversions including the most divergent components (Supplementary Table S8; Supplementary Figure S2). Amino sugar and nucleotide sugar metabolism was interacted with the other four pathways (Figure 5). The majority of genes and substances in these five pathways were significantly downregulated in the BPH that fed on RH varieties, which indicated that metabolite accumulation was specifically regulated by the DEGs. The above results showed that feeding on the RH-resistant rice variety significantly inhibited the anabolism of sterols and vitamins (i.e., ascorbate) and energy substances (i.e., sucrose and galactose), which was consistent with the bioassay results.

Among all related differential factors, the downregulated metabolite D-Glucose-1P was involved in amino sugar and nucleotide sugar metabolism and starch and sucrose metabolism, whereas the downregulated gene LOC111062462 (α-glucosidase) was involved in starch and sucrose metabolism and galactose metabolism pathways. LOC111058748 (UDP-glucosyltransferase) was involved in ascorbate and aldarate metabolism and pentose and glucuronate interconversions, indicating these reduced functions in the regulatory network of BPH that fed on RH-resistant rice varieties. In addition, we observed increased levels of the glycerol and xylitol, the product of fat metabolism and the intermediate product of carbohydrate metabolism, respectively. Overall, network analysis showed that the DEGs were strongly positively correlated with certain metabolites, especially in the metabolism of lipids and carbohydrates in BPH.

## Discussion

BPH is the most dangerous pest of rice (Guo et al., 2018; Du et al., 2020; Shi et al., 2021). The use of insect-resistant rice varieties is regarded as the most economical, effective, eco-friendly, and sustainable strategy for controlling BPH (Zhang et al., 2022). However, over time, the adaptation levels of BPH adaptation to insect-resistant rice plants have developed rapidly, resulting in the establishment of a strongly novel BPH virulent population (Ji et al., 2013; Kobayashi, 2016; Huang et al., 2017; Ji et al., 2017). Therefore, understanding the complex BPH-plant interactions is important for developing environment-friendly BPH-combating strategies. Previous studies have mainly focused on the single omics of the effects of insect-resistant rice varieties on BPH (Liu et al., 2010; Peng et al., 2016; Liu et al., 2017; Yue et al., 2019), paying less attention to the integrated analysis of the transcriptome and metabolome.

In the current study, we investigated fitness loss and focused on transcriptomic and metabolomic changes to determine the effects of insect-resistant rice varieties on BPH. After consuming RH rice types, a decrease in fertility, fat content, and significant changes in the transcriptomic and metabolic profiles of BPH were detected. Across all samples, a total of 481 metabolites were identified, including 145 DAMs. The DAMs were predominantly abundant in amino acid metabolism and carbohydrate metabolism, such as glycolysis or gluconeogenesis, starch and sucrose metabolism, as well as D-arginine and D-ornithine metabolism. This suppressed energy metabolism in BPH that fed on RH plants. Most DAMs are amino acids and derivatives, organic amines, sterols, vitamins (such as glucuronic acid), and fatty acids which indicates that the synthesis of these metabolites was suppressed. In addition, glycerol and xylitol were significantly increased in BPH that fed on RH plants, suggesting the accelerated decomposition of energy storage substances. In summary, RH rice varieties could reduce BPH fitness by affecting its energy supply and nutritional requirements. Transcriptomic analysis revealed that BPH that fed on RH plants showed significant changes in physiology and gene expression, and 333 upregulated and 486 downregulated genes were detected compared with those detected for BPH that fed on TN1 plants. These genes were mainly involved in carbohydrate metabolism, energy metabolism, amino acid metabolism, hormone synthesis and vitamin metabolism pathways. Finally, a combined analysis of the metabolome and transcriptome was carried out, and it was established that energy material metabolism was significantly affected.

Plant nutrients, including sugars, amino acids, vitamins, and sterols, provide essential energy for BPH growth, maintenance, reproduction, flight, and storage (Chen et al., 2012). Trehalose is the main blood sugar and energy source in insect bodies (Gu et al., 2009). In the current investigation, we found the content of trehalose in BPH that fed on RH plants were significantly decreased, which is consistent with previous report that BPH feeding on resistant rice plants significantly lower the trehalose content (Yang et al., 2016). These results indicated that trehalose may play important roles in the interaction between insect-resistant rice and BPH, while the regulatory mechanism is still unclear and needs further study from the perspective of trehalose anabolic pathway. Inhospitable environments are energetically demanding for insects. Previous metabolic analyses of BPH that fed on IR56 showed suppressed expression of energy metabolism pathways [including glycolysis and the citric acid (TCA) cycle] (Yue et al., 2019). Our data also showed that the TCA cycle was significantly affected (Supplementary Table S5), and the pyruvate carboxylase coding gene, a key enzyme in gluconeogenesis, and the ATP-citrate synthase coding gene, an enzyme that controls the entry of the TCA cycle, were both downregulated, as reported by Fujita et al. (2013). In addition, we found that the glycerol and xylitol levels increased. Therefore, we deduced that feeding on RH-resistant rice significantly inhibited the TCA cycle of BPH and promoted the decomposition of energy storage substances, first carbohydrates then fats, resulting in an increase in the intermediate product of carbohydrate metabolism and free fatty acid (and glycerol) levels in BPH. However, contrary to our results, Peng et al. (2016) found that feeding on insect-resistant rice YHY15 which contains *Bph15* for 48 h, significantly upregulated the intermediates (succinate and malate) expression of the TCA cycle pathway in BPH compared with feeding on the TN1 rice variety. Liu et al. (2017) also found that the level of trehalose in the BPH feeding on YHY15 for 24 h was raised, compared with BPH feeding on the susceptible plants. This may be due to the different resistance mechanisms of the different insect-resistant rice varieties to BPH. In our opinion, both RH and IR56 contain multiple resistance genes, one of which is *Bph3*, Os*LecRK1*- Os*LecRK3* probably acting as a receptor to recognize BPH related molecules located outside rice cells, sensing the feeding of BPH and initiate resistance response, and thus constituting the first barrier for rice against BPH (Liu et al., 2015). Meanwhile, IR56 also contains *Bph32* resistance gene encoding an unknown SCR domain containing-protein, which is expressed at a high level in the leaf sheath of rice plants and may inhibit the feeding of BPH (Ren et al., 2016). In general, RH and IR56 may affect the BPH energy metabolism by affecting its feeding. YHY15 only contains *Bph15*, which is considered to function in a way same as *Bph3* (Du et al., 2018). This suggests that the different metabolic effects of different resistant rice lines on the BPH metabolome may be due to the number of resistance genes, and relevant reports have confirmed this (Qiu et al., 2012; Hu et al., 2013). Another reason for this phenomenon may resulted from BPH adaptation to the adverse environment with their increased hopping frequency to ingest more sap together with contributions from yeast-like symbionts in BPHs feeding on YHY15 (Hao et al., 2008; Liu et al., 2017).

In addition to carbohydrate metabolism, the amino acid metabolism pathways of arginine, ornithine, proline, phenylalanine, aspartate, cysteine, and methionine in BPH that fed on RH plants were also significantly changed. Amino acids are the raw material for protein synthesis and also play a crucial role in fundamental insect processes (Hargrove, 1976; Andersen, 2007; Holmstrup and Bayley, 2013; Novoselov et al., 2015). At the transcriptomic and metabolic levels, cysteine and methionine metabolism pathways were both significantly affected (Supplementary Table S5; Supplementary Table S7). Previous studies have revealed that a lack of methionine can significantly prolong BPH nymph development duration and limit the emergence rate (Fu et al., 2001b). Matthews et al. (2020) predicted that bacterial cysteine and methionine metabolism genes could influence the longevity of *Drosophila melanogaster*. Therefore, we speculate that the decreased ecological adaptability of BPH that fed on RH plants may be related to abnormal cysteine and methionine metabolism. In addition, our transcriptomic results showed that vitamin B6 metabolism and vitamin digestion and absorption pathways (Figures 3B, 4C) decreased significantly, and bacteria benefiting from the host insect’s vitamin biosynthesis are a common phenomenon (Bauer et al., 2014; Salem et al., 2014; Ju et al., 2020). The essential amino acids required for hemipteran insects are mainly synthesized with the aid of endosymbionts (Douglas, 1998; Pang et al., 2012; Ferrater et al., 2013) because rice phloem sap, the sole food source of BPH, cannot provide sufficient nutrients (Hayashi and Chino, 1990). We further speculate that BPH feeding on RH plants has an impact on symbiotic bacteria *in vivo* and then affects the synthesis and metabolism of amino acids. The metabolomic analysis also revealed the significant change in the arginine and ornithine metabolism and arginine and proline metabolism pathway (Figure 4). Compared with that in BPH that fed on TN1 plants, the content of arginine in BPH that fed on IR26 resistant variety was significantly altered (Xu et al., 2008). Many insects have nutritional requirements for arginine (Reddy and Campbell, 1977). This suggests that the arginine metabolism of BPH is greatly affected by RH, which affects the nutritional level of BPH and reduces its ecological fitness. In addition, the current study found that BPH that fed on RH plants reduced the metabolism of several aliphatic amino acids (L-serine, D-glutamate, glycine, L-asparagine, and beta alanine), which are closely associated with fatty acid metabolism.

Fatty acids serve as energy stores in insects (Stanley-Samuelson et al., 1988), and are essential nutrients that play important roles in innate insect immunity (Stanley et al., 2012). In the metabolome obtained herein, we discovered that BPH that fed on RH plants tended to contain fewer fatty acids (Supplementary Table S6), which was consistent with previous studies (Alagar et al., 2007; Yue et al., 2019; Cui et al., 2020). In contrast, the concentrations of some organic acids, amides, and derivatives of fatty acids were significantly higher in BPH that fed on RH plants than in the controls. This indicates that the increased transformation of fatty acids to their respective derivatives adversely affected the physiological activity of BPH. Consequently, the reduction in fatty acids may also potentially account for the reduced ecological adaptability of BPH observed in this study.

## Conclusion

In summary, there were significant differences in ecological fitness, like survival rate, adult weight, the number of eggs laid per female, fat content and honeydew secretion between BPH reared on RH and TN1 varieties. 819 DEGs and 145 DAMs were identified and these factors mainly enriched in energy metabolism, amino acid metabolism and vitamin metabolism pathways. Combined analyses of transcriptome and metabolome showed that pathways, including starch, sucrose, and galactose metabolism, were altered. The present study provides a comprehensive overview to better understand how insect-resistant rice cultivars combat BPH infestation.

## Data Availability

The transcriptome data generated in this study are deposited in the NCBI SRA database under BioProject PRJNA869778. The names of the repository/repositories and accession number(s) can be found in the article/Supplementary Material, further inquiries can be directed to the corresponding author.

## Supplementary material

SUPPLEMENTARY FIGURE S1 Orthogonal partial least squares-discrimination analysis (OPLS-DA) plots of metabolite composition of samples from BPH reared on RH and TN1 varieties. Each symbol represents a sample; Different symbol shapes denote different groups. The circle line indicates the 95% confidence interval (Hotelling’s T-squared ellipse).

SUPPLEMENTARY FIGURE S2 Pathways of Galactose metabolism, **(A)** Ascorbate and aldarate metabolism **(B)**, Pentose and glucuronate interconversions **(C)**, Starch and sucrose metabolism **(D)** and Amino sugar and nucleotide sugar metabolism **(E)** affected by different rice varieties. Red indicates that the gene/compound is upregulated, blue indicates that the gene/compound is downregulated.
