# Supplementary figures and images for: Transcriptome and metabolome analyses reveal the responses of brown planthoppers to RH resistant rice cultivar
Source: Front Physiol. 2022 Sep 16;13:1018470. doi: 10.3389/fphys.2022.1018470 (PMC9523508; doi:10.3389/fphys.2022.1018470)

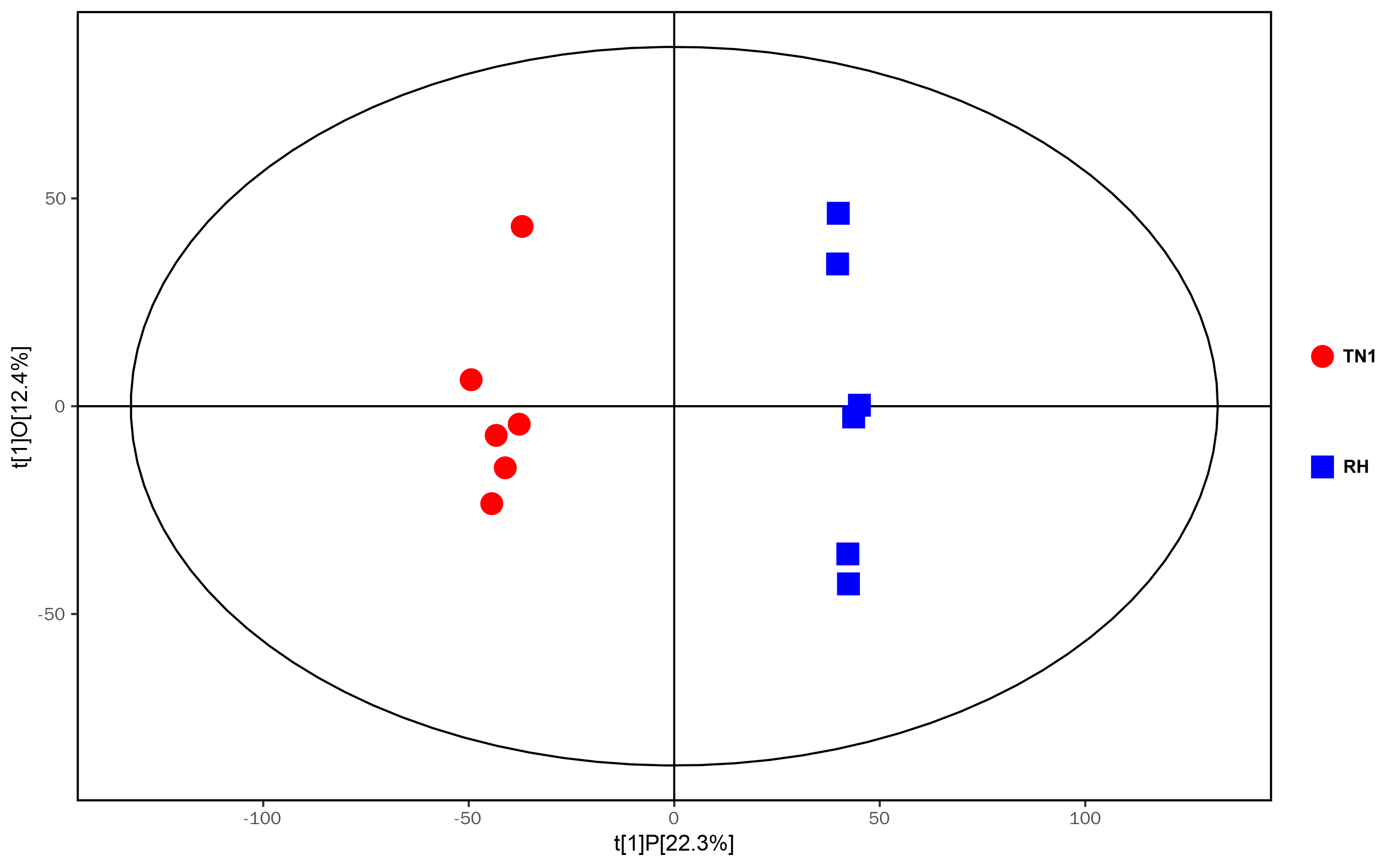

Supplement: Supplementary file 4 [file Image1.TIF]
